# Ubiquitination-mediated degradation of TRDMT1 regulates homologous recombination and therapeutic response

**DOI:** 10.1093/narcan/zcab010

**Published:** 2021-03-22

**Authors:** Xiaolan Zhu, Xiangyu Wang, Wei Yan, Haibo Yang, Yufei Xiang, Fengping Lv, Yi Shi, Hong-yu Li, Li Lan

**Affiliations:** Massachusetts General Hospital Cancer Center, Harvard Medical School, Charlestown, MA 02129, USA; Department of Radiation Oncology, Massachusetts General Hospital, Harvard Medical School, Boston, MA 02115, USA; Massachusetts General Hospital Cancer Center, Harvard Medical School, Charlestown, MA 02129, USA; Department of Radiation Oncology, Massachusetts General Hospital, Harvard Medical School, Boston, MA 02115, USA; Department of Pharmaceutical Sciences, College of Pharmacy, University of Arkansas for Medical Sciences, Little Rock, Arkansas 72205, USA; Massachusetts General Hospital Cancer Center, Harvard Medical School, Charlestown, MA 02129, USA; Department of Radiation Oncology, Massachusetts General Hospital, Harvard Medical School, Boston, MA 02115, USA; Department of Cell Biology, University of Pittsburgh, 3501 fifth Ave., Pittsburgh, PA 15260, USA; Department of Pharmaceutical Sciences, College of Pharmacy, University of Arkansas for Medical Sciences, Little Rock, Arkansas 72205, USA; Department of Cell Biology, University of Pittsburgh, 3501 fifth Ave., Pittsburgh, PA 15260, USA; Department of Pharmaceutical Sciences, College of Pharmacy, University of Arkansas for Medical Sciences, Little Rock, Arkansas 72205, USA; Massachusetts General Hospital Cancer Center, Harvard Medical School, Charlestown, MA 02129, USA; Department of Radiation Oncology, Massachusetts General Hospital, Harvard Medical School, Boston, MA 02115, USA

## Abstract

The RNA methyltransferase TRDMT1 has recently emerged as a key regulator of homologous recombination (HR) in the transcribed regions of the genome, but how it is regulated and its relevance in cancer remain unknown. Here, we identified that TRDMT1 is poly-ubiquitinated at K251 by the E3 ligase TRIM28, removing TRDMT1 from DNA damage sites and allowing completion of HR. Interestingly, K251 is adjacent to G155 in the 3D structure, and the G155V mutation leads to hyper ubiquitination of TRDMT1, reduced TRDMT1 levels and impaired HR. Accordingly, a TRDMT1 G155V mutation in an ovarian cancer super responder to platinum treatment. Cells expressing TRDMT1-G155V are sensitive to cisplatin *in vitro* and *in vivo*. In contrast, high expression of TRDMT1 in patients with ovarian cancer correlates with platinum resistance. A potent TRDMT1 inhibitor resensitizes TRDMT1-high tumor cells to cisplatin. These results suggest that TRDMT1 is a promising therapeutic target to sensitize ovarian tumors to platinum therapy.

## INTRODUCTION

Recent work has identified and mapped a range of post-transcriptional modifications in mRNA, including methylation of the N6 and N1 positions in adenine, pseudouridylation and methylation of carbon 5 in cytosine (m^5^C) (1,2). Increased mRNA:m^5^C was detected in 5-azacytidine (5-AZA)-resistant clinical AML/MDS cells (3). However, knowledge about RNA:m^5^C methyltransferases (RCMTs) and the consequences of RNA m^5^C modification is still extremely limited.

tRNA-aspartic acid methyltransferase1 (TRDMT1), known as DNA methyltransferase 2 (DNMT2), was identified as a highly conserved cytosine-C5 methyltransferase that introduced the C38 methylation of tRNA-Asp in many species (4–8). TRDMT1 does not show methyltransferase activities on DNA but was recently shown to contribute to mRNAs methylation in addition to tRNA (9–11). TRDMT1 is essential for oxidative stress and heat shock-induced tRNA cleavage (12) and is involved in the resistance to 5-AZA by directly binding hnRNPK (3,13). We used KillerRed (KR), which releases O^−^_2_ in a light-inducible manner to cause single-strand breaks and double-strand breaks (DSBs), to induce DNA damage at specific sites in the genome and locally activate DNA repair pathways (14,15). We found DNA damage-induced and TRDMT1 mediated-mRNA m^5^C is required for the efficient repair of DSBs by promoting homologous recombination (HR) (16). These new findings indicate an important role of TRDMT1 in cell survival after DNA damage, motivating us to investigate the function of TRDMT1-mediated RNA m^5^C modification in cancer therapy. Of note, how TRDMT1 is regulated in cells after damage is still not understood. The cytosine analogs azacytidine and decitabine have been developed as DNA methyltransferases (DNMTs) inhibitors for cancer therapy, but their use was limited owing to poor bioavailability and toxicities (17). Although selective inhibitors have been developed for the protein lysine/arginine methyltransferases (PKMTs/PRMTs) and DNMTs (18–20); no known inhibitors of RNA methyltransferases (RNMTs) beyond the reaction product S-Adenosylhomocysteine (AdoHcy, or SAH) and the universal nucleoside analog sinefungin (21).

In this study, we explored the relevance of TRDMT1 in the treatment response of ovarian cancers. Using mass spectrometry analysis, we identified that the TRDMT1 is highly polyubiquitinated at Lysine 251 (K251) via the E3 ligase TRIM28, also named KAP-1. K251 is adjacent to G155 in the 3D protein structure. Interestingly, G155V mutation of TRDMT1 leads to excessive TRDMT1 degradation, and subsequent loss of TRDMT1 function leads to inefficient repair and contributes to increased DNA damage sensitivity of cancer cells. Indeed, TRDMT1 G155V mutation identified in a patient with ovarian cancer is sensitive to platinum therapy. Given the crucial role of TDRMT1 in the DNA damage response of cancer cells, we developed a potent inhibitor of TRDMT1 and showed that it sensitizes cancer cells to DNA-damaging agents, thereby opening a new avenue to targeting RNMTs in cancer therapy.

## MATERIALS AND METHODS

### Cell culture and transfection

U2OS, 293, MCF-7, SKOV3, HCC-1954 and HCC-1937 cells were cultured in DMEM with 10% (v/v) FBS in a controlled humidified atmosphere containing 5% CO_2_ at 37°C. The U2OS-TRE cells used for the DART system were derived from the U2OS-263 cell line and the construction of U2OS TRDMT1 knockout (KO) cells has been described previously (16). Lipofectamine 2000 and Lipofectamine RNAiMax (Invitrogen) were used for plasmid and siRNA transfection, respectively, following a standard protocol. TRDMT1 siRNA was purchased from Invitrogen (siRNA ID: s4219, Cat#: 4392420). TRIM28 siRNA sequence is GCGGAAAUGUGAGCGUGUACACGCUCACAUUUCCG.

### Plasmids

The TA-KR in pBroad3 plasmids were constructed as previously described (15). The GFP spark-TRDMT1 was purchased from Sino biological (HG11224-ACG). The cDNAs of WT and mutated TRDMT1 (C79A, G155V, K251R, K122R, G155V/K251R) were then sub-cloned to the EGFP-C3 plasmid (Clontech), linked by EcoRI and BamHI for imaging experiments. Primers are shown in Supplementary Table S2. The construction of NLS-GFP-RAD52 and NLS-I-SceI plasmids have been described previously (22).

### Cell survival assay

Approximately 500 U2OS/TRDMT1 KO U2OS/MCF-7/HCC1954/HCC1937 cells and TRDMT1 siRNA or GFP-TRDMT1 WT/G155V/K251R transfected cells were seeded in 6 cm dish with or without TRDMT1 inhibitors (2.5 μM/l). Six hours after seeding, cells were exposed to cisplatin (1134357, Sigma, 1 μM/l), ATMi (KU55933, C9867, Sigma, 10 μM/l), ATRi (AZ20,S7050, Selleck, 1 μM/l) or PARPi (Olaparib,AZD2461, Sigma, 1 μM/l) at indicated concentration or ionizing radiation (IR). After 7–14 days, colonies were fixed and stained with 0.3% crystal violet in methanol, and the number of colonies was counted manually.

### Microscopy and activation of KillerRed

The Olympus FV1000 confocal microscopy system (Cat. F10PRDMYR-1, Olympus) and FV1000 software were used for the acquisition of images. Cells were cultured in 35 mm glass-bottom dishes (MatTek, P35GC-1.5–14-C) before observation. Activation of KR in bulky cells was completed by exposing cells to a 15 W Sylvania cool white fluorescent bulb for 25 min in a stage UVP (Uvland, CA). The intensity was measured by ImageJ 1.52i software.

### Immunoprecipitation and LC/MS analysis of GFP-TRDMT1

GFP-TRDMT1 stably expressed Flp-in 293 cells were treated with or without 2 mM H_2_O_2_ for 3 h before harvest as damaged and undamaged conditions. Wild-type 293 cells were used as control. The cell noodles were made in liquid nitrogen and then cryogenically milled into micron-sized particles to maximize the efficiency of solvent extraction of proteins. Two lysis buffer (20 mM HEPES, 0.5% TritonX-100 and protease inhibitors) with different salt concentrations (150 or 300 mM NaCl) were used to capture the protein interactions while preserving the extraction efficiency (23). Affinity purification of GFP-TRDMT1 from the whole cell lysates was carried out by an anti-GFP nanobody (24) coupled to magnetic dynabeads (Thermo, Cat# 14302D). After protein reduction and alkylation, the immunoprecipitation samples under each condition were run on a 4–12% SDS-PAGE gel using a short gradient. The whole region of each sample was cut and in-gel digested with trypsin as previously described (25). After proteolysis, the peptide mixtures were desalted and analyzed with a nano-LC 1200 coupled to a Q Exactive™ Orbitrap™ mass spectrometer (Thermo Fisher). The peptides were loaded onto a picochip column (C18, 3  μm particle size, 300 Å pore size, 50 μm × 10.5 cm; New Objective) and eluted using a 60 min LC gradient: 7% B–12% B, 0–3 min; 12% B–40% B, 3–50 min; 40% B–100% B, 50–53 min; 100% B, 53–60 min; mobile phase A consisted of 0.1% formic acid (FA) in LC/MS water, and mobile phase B consisted of 0.1% FA in 100% acetonitrile. The QE instrument was operated in the data-dependent mode, where the top 10 most abundant ions (mass range 350–1500, charge state  2–6) were fragmented by high-energy collisional dissociation (normalized collision energy 30). The target resolution was 60 000 for MS and 7500 for MS/MS analyses. The quadrupole isolation window was 2.0 Th and the maximum injection time for MS/MS was set at 100 ms. After MS analysis, the data were searched by Maxquant (26) for identification and label-free quantification. The mass accuracy was specified as 10 and 20 ppm for MS and MS/MS, respectively. Other search parameters included cysteine carbamidomethylation as a fixed modification and methionine oxidation as a variable modification. A maximum of three trypsin missed-cleavage sites was allowed.

For western blot analysis, proteins from cells or tissues were separated by SDS-PAGE and then transferred to polyvinylidene difluoride membranes (PVDF; Bio-Rad, USA). The membranes were blocked with 5% non-fat milk in PBS and probed with antibodies against TRDMT1 (sc-365001, Santa Cruz Biotechnology,1:400), TRIM28 (ab10484, Abcam, 1:1000), Ub (sc-8017, Santa Cruz Biotechnology, 1:500), Tubulin (12004165, Bio-Rad, 1:1000), β-actin (8H10D10, Cell Signaling Technology, 1:10 000). After primary antibody incubation at 4°C overnight and secondary antibody incubation at room temperature for 1 h, the membranes were washed three times in 0.1% PBST. Chemiluminescent HRP substrate was purchased from Millipore (Catalog#: WBKLS0500). Images were taken in the BIO-RAD Universal Hood II machine with corresponding ImageLab software.

### Dot blot assay

Total poly(A)+ mRNA from U2OS-TRE cells was purified with Dynabeads™ mRNA DIRECT™ Purification Kit (ThermoFisher Scientific, Catalog#: 61011). The amount of mRNA from different samples was diluted to the same concentration using 10 mM Tris-HCl from the kit. The mRNA solutions were loaded onto a positive-charged Nylon66 membrane (Biodyne B transfer membrane, 0.45 μm, 60209) and 1200 μJ was applied twice to the membrane in 1 min (UV Stratalinker 2400). After primary antibody (1:100) incubation at 4°C overnight and secondary antibody (1:10 000) incubation at room temperature for 1 h, the membrane was washed three times in 0.02% PBST for 10 min each. The following steps were the same as in the western blot. The membrane was stained with 0.1% methylene blue (SIGMA-ALDRICH, Catalog#: M9140–25G) in 0.5 M sodium acetate.

### Immunoassays and m^5^C staining

Cells for immunofluorescence observation were fixed in 4% PFA (Affymetrix, 19943 1 LT) at room temperature for 15 min and further treated with 0.2% triton for 10 min. They were then blocked by 3% BSA (SIGMA, A-7030) at room temperature for 1 h. Primary antibody for RAD51 (ab63801, Abcam, 1:200)), RAD52 (sc-365341, Santa Cruz, CA, USA, 1:500), TRIM28 (ab10484, Abcam, 1:500) and γH2AX ser139 (JBW301, 05–636, EMD Millipore, 1:200) were incubated with cells at 4°C overnight. After washing with 0.05% PBS-Tween (PBST), the cells were incubated with secondary antibody for 1 h at room temperature.

For m^5^C staining using the heat method, the cells or tissues were fixed and permeabilized in a 35 mm glass-bottom dish using a standard protocol, incubated in buffer (10 mM Tris-HCl, 2 mM EDTA, pH 9), and steamed on a 95°C heating block for 20 min to expose the antigen. The dish was cooled, washed three times with PBS and blocked using 5% BSA in 0.1% PBST for 0.5 h at room temperature. Primary antibody for m^5^C (33D3, ab10805, Abcam) and secondary antibody were diluted in the same buffer (5% BSA in 0.1% PBST) and followed the standard IF protocol. This protocol is modified from the classic heat-induced antigen retrieval method for paraformaldehyde-fixed tissues using Tris-EDTA buffer.

### Animal assay

SKOV3 cells (1.0 × 10^6^) were transfected with LV-TRDMT1-RNAi (gcAGAAGA AATTCACAGGAAA) or LV-NC-RNAi and injected intraperitoneally into female nude mice that were then randomly divided into four groups (six animals per condition). When mice developed palpable tumors (about a week after injection), cDDP (5 mg/kg) or saline was then injected into the center of the xenograft tumors twice per week for 3 consecutive weeks. On day 28, mice were sacrificed, and tumors were harvested. Terminal deoxynucleotidyl transferase dUTP nick end labeling (TUNEL) (In Situ Cell Death Detection Kit, Fluorescein, 11684795910, Roche, Basel, Switzerland) was used to assess tissue apoptosis according to the instructions. All animal experiments were approved by and conducted according to the guidelines established by the Institutional Animal Care and Use Committee at the University of Pittsburgh. With the approval of the University of Pittsburgh, serous ovarian cancer samples from patients with FIGO stage IIIC or IV (*n* = 38) were collected from tissue banks. All patients were treated with the standard care of platinum-based therapy after surgery, and informed consent was obtained from all patients. PFS was calculated from the time of surgery to the time of progression or recurrence. Platinum resistance or platinum sensitivity was defined by relapse or progression within 6 months or 6 months after the last platinum-based chemotherapy, respectively. Clinical and pathological features are described in Supplementary Table S1.

### Immunohistochemistry and scoring

Tumors were fixed, embedded in paraffins and sectioned into 4 μm thickness. After deparaffinization and rehydration, sections were blocked and incubated with antibody against TRDMT1(sc-365001, Santa Cruz, CA, USA,1:200), Ki-67 (sc-23900, Santa Cruz,1:200), and then detected using the Dako Envision two-step method of immunohistochemistry (Carpinteria, CA, USA). All IHC staining was scored independently by two pathologists. We divided the positive staining results into 0–4 categories as following: 0: <5%; 1: 6–25%; 2: 26–50%; 3: 51–75%; and 4: >76% staining.

### NHEJ and HR assay

Using a previously described method (27), U2OS (DR-GFP) and U2OS (EJ5-GFP) cells were seeded into six-well plates and treated with indicated siRNAs and inhibitors after 16–24 h of seeding. About 1 μg of I-SceI vector was transfected into siRNA or inhibitors pretreated cells using Lipofectamine 2000 (Invitrogen). Cells were then harvested by trypsinization and washed with PBS 48 h after I-SCEI transfection. The GFP signal arising from the recombination (HR) or nonhomologous end joining (NHEJ) events was measured by flow cytometry.

### Synthesis of YW-1842

The synthesis of YW-1842 is illustrated in Supplementary Scheme S1. Starting from the commercially available 2,4-dichloro-6,7-dimethoxyquinazoline, YW-1842 is obtained through a sequential Suzuki-coupling reaction and microwave-assisted nucleophilic substitution reaction. YW-1842 is purified to be >95% and characterized by NMR and LCMS. Detailed synthetic procedures and analytical data are reported in the Supplementary Information (Supplementary Figures S4 and S5).

### Statistical analysis

The data were presented as mean ± SD from at least three independent experiments. Comparisons between each group were calculated using Student’s *t*-test, two-tailed Fisher’s exact test method of summing small *P*-values, one- and two-way analysis of variance, and Bonferroni’s multiple comparison test as appropriate. A value of *P*< 0.05 was considered significant. GraphPad Prism version 7 was used for graphics (GraphPad Software, San Diego, CA, USA).

## RESULTS

### TRDMT1 is regulated by polyubiquitination and the TRDMT1^G155V^ mutant is highly poly-ubiquitinated

**Figure 1.**
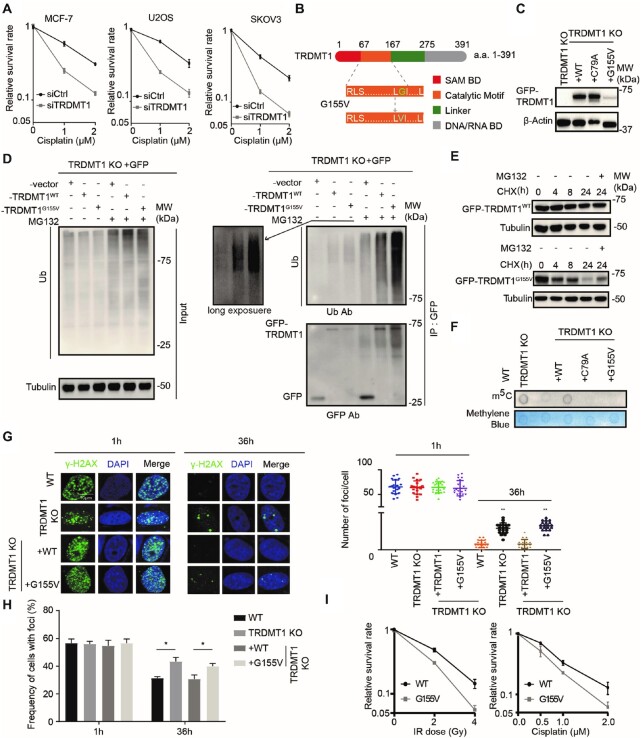
TRDMT1^G155V^ is highly ubiquitinated in cells. (**A**) The survival rate of MCF-7, U2OS and SKOV3 cells with siCtrl or siTRDMT1 and indicated dose of cisplatin (*n* = 3, mean ± SD). (**B**) Schematic diagram of TRDMT1 and TRDMT1^G155V^ mutant. (**C**) WB ofTRDMT1 in GFP-tagged TRDMT1^WT^, TRDMT1^C79A^ orTRDMT1^G155V^ stably expressed TRDMT1 KO 293 cells. (**D**) The ubiquitination level of TRDMT1 in GFP-vector TRDMT1^WT^or GFP-TRDMT1^G155V^transfected TRDMT1 KO 293 cells. Indicated cell lines were treated with or without 20 μM MG132 for 6 h before protein extraction. (**E**) WB of GFP-TRDMT1 in TRDMT1^WT^or GFP-TRDMT1^G155V^ transfected TRDMT1 KO U2OS cells treated with 100 μg/ml CHX with or without 20 μM MG132 for the indicated time. (**F**) Dot blot analysis of m^5^C in mRNA of TRDMT1 KO 293 cells with stable expression of GFP-tagged TRDMT1^WT^, TRDMT1^C79A^ or TRDMT1^G155V^. (**G–H**) γ-H2AX staining in GFP-tagged TRDMT1^WT^ or TRDMT1^G155V^transfected TRDMT1 KO U2OS-TRE cells. Cells were irradiated with 6 Gy IR and stained with γ-H2AX at the indicated time point, numbers of γ-H2AX foci in each cell (G) and the frequency of 100 cells with γ-H2AX foci (H) were counted in each experiment (*n* = 3, mean ± SD). (**I**) The survival rate of the cells after IR or Cisplatin in GFP-tagged TRDMT1^WT^ or TRDMT1^G155V^transfectedTRDMT1 KO U2OS-TRE cells (*n* = 3, mean ± SD). Statistical analysis was done with the Student's *t*-test, *: *P*< 0.05; **: *P*< 0.01; ***: *P*< 0.001; ****: *P*< 0.0001 for all the figures in the paper.

TRDMT1 was found consistently up-regulated in hundreds of tumor samples listed in the COSMIC database and >90 somatic mutations in TRDMT1 have been identified in tumors of various tissue types (28), while little is known regarding how its mutations influence tumorigenesis and affect treatment response in cancer. Our previous study indicated that TRDMT1-mediated mRNA m^5^C formation promoted HR in the transcriptionally active regions of the genome (16). The role of TRDMT1 raises the possibility that TRDMT1-regulated mRNA m^5^C modification might be involved in resistance to other damage agents that induce HR to repair damage. Cisplatin induces DNA cross-linking and subsequent DSBs and triggers HR (29,30). Indeed, we observed enhanced sensitivities of cells treated with siTRDMT1 to cisplatin in multiple cell lines including U2OS, breast cancer cell line MCF-7 and ovarian cancer cell line SKOV3 (Figure 1A), confirming that downregulation of TRDMT1 increases the sensitivity of tumor cells to Cisplatin.

G155 is a hotspot of somatic cancer mutations, which locates on the back side, right in front of motif VIII of TRDMT1 (Figure 1B). The Cancer Genome Atlas (TCGA)-13–0720 reveals that a patient bearing TRDMT1^G155V^ mutation is a super responder to the primary platinum treatment, displaying a complete response to cisplatin treatment and exhibiting a better prognosis (28). Eventually, the patient had almost a year longer overall survival time compared with the patients at the same clinical stage (stage III). As known, cancer mutations may reduce the stability of the protein, alter its localization or affect the methylation activity of RNA substrates (28,31). Comparing with stably expressed TRDMT1^WT^ and TRDMT1^C79A,^ a methyltransferase catalytic deficiency mutant, the protein level of TRDMT1 was consistently low in TRDMT1^G155V^ stably expressed 293 TRDMT1 KO cell line (Figure 1C). To understand the low expression of TRDMT1 is due to protein instability or degradation, we examined the ubiquitination level of TRDMT1^WT^ or TRDMT1^G155V^. GFP, GFP-WT and GFP-G155V were IPed with anti-GFP antibody in the absence or presence of MG132. In the absence of MG132, less GFP-G155V than GFP-WT was detected at the ‘unmodified’ position (∼70 kDa) (Figure 1D, left). Furthermore, in the presence of MG132, more GFP-G155V than GFP-WT was detected in the ‘ubiquitinated’ smears (>70 kDa) (Figure 1D, right). These observations suggest that G155V is more ubiquitinated and less stable than WT. Moreover, degradation of TRDMT1^G155V^ was faster than TRDMT1^WT^ after treatment of a protein synthesis inhibitor, cycloheximide (CHX) (Figure 1E). Degradation of TRDMT1 is prevented by MG132, suggesting that this is a proteosome-dependent degradation (Figure 1E). TRDMT1 is an mRNA m5C writer at sites of transcribed damage, our previous study indicates the m^5^C in mRNA is required for damage removal in the nucleus and drug-resistance of cells. We, therefore, measured the m^5^C level of mRNA. The level of m^5^C in mRNA extraction was decreased in TRDMT1^G155V^stably expressed TRDMT1 KO 293 cell line compared to WT (Figure 1F). It is not surprising that the γH2AX clearance was delayed at sites of damage (Figure 1G and H) and the survival rates of both cisplatin- and IR-treated cells were decreased in TRDMT1^G155V^compared to TRDMT1^WT^ expressed TRDMT1 KO cells (Figure 1I). These results indicate that the stability of TRDMT1 in cells is regulated by ubiquitination, and importantly, insufficient repair capacity of G155V-mutated TRDMT1 contributes to cell death after damage.

### TRIM28 promotes DNA damage-induced TRDMT1 ubiquitination at transcriptionally active sites

**Figure 2.**
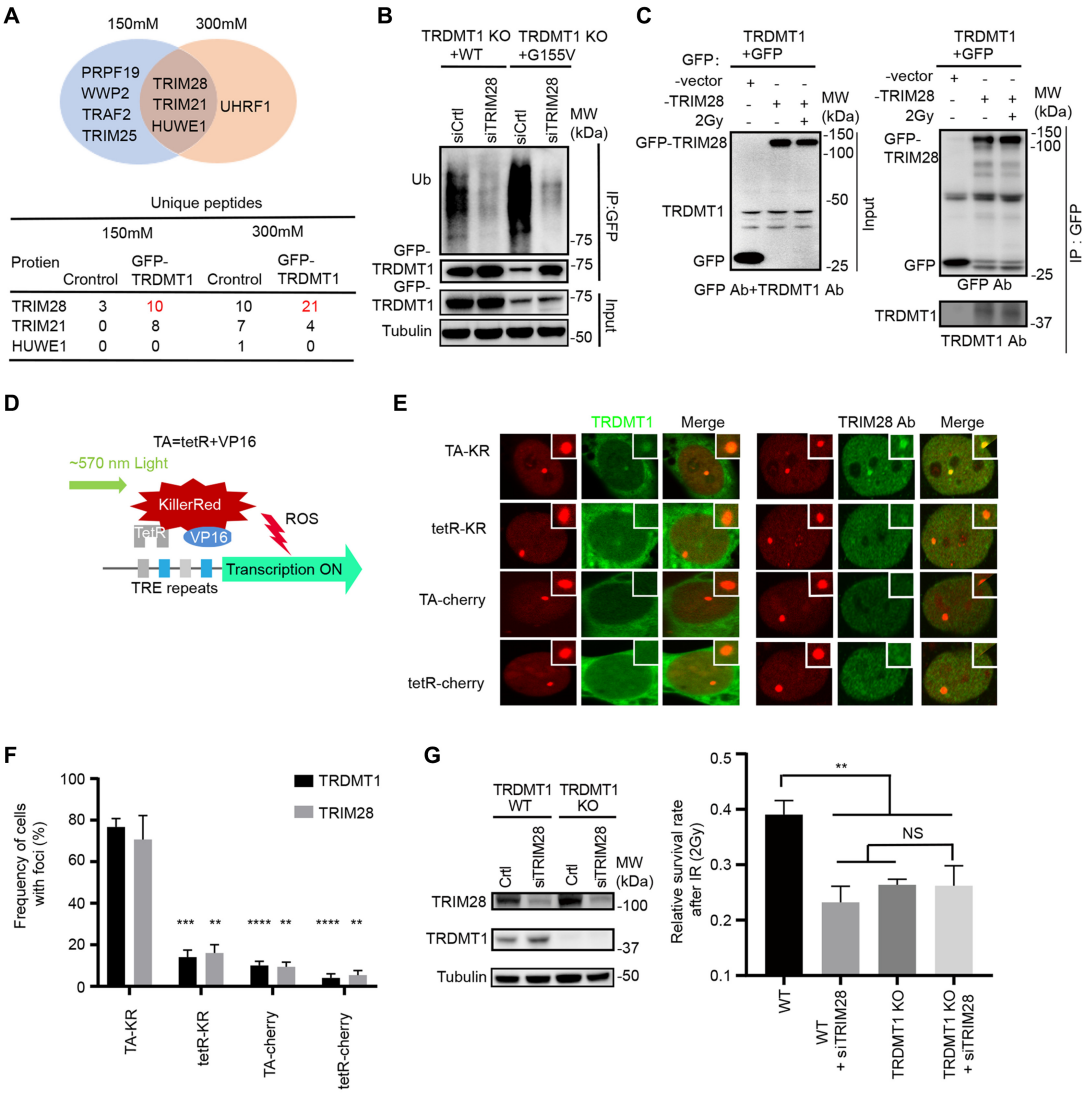
TRDMT1 is ubiquitinated by E3 ligase TRIM28 at sites of DNA damage. (**A**) GFP-TRDMT1 stably expressed 293 cells were immunoprecipitated by anti-GFP and washed with sodium chloride concentrations at 150 or 300 mM. E3 ligases identified from mass spectrometry by pulling down by GFP-TRDMT1 under each wash condition and numbers of unique peptides of E3 ligases are indicated. (**B**) 293 TRDMT1 KO cells stably expressing GFP-tagged TRDMT^WT^ and TRDMT1^G155V^were pulled with anti-GFP and immunoblotted with anti-ubiquitin. Cells were treated with 20 μM MG132 for 6 h before pulling down. (**C**) IP of GFP-TRIM28 and TRDMT1. GFP-vector or GFP-TRIM28 transfected U2OS-TRE cells were treated with or without IR. GFP-vector or GFP-TRIM28 was pulled down by anti-GFP and immunoblotted with anti-TRDMT1. (**D**) Schematic diagram of KillerRed (KR)-mediated damage at genomic loci in U2OS-TRE cells. (**E**) U2OS-TRE cells co-transfected TA-KR/TA-Cherry/tetR-KR/tetR-Cherry with or without GFP-TRDMT1 plasmid were exposed to light for 30 min for KR activation and allowed to recover for 30 min before fixation. Recruitment of TRIM28 with anti-TRIM28 antibody at each indicated site is shown. (**F**) Quantification of the frequency of cells in 50 cells with GFP-TRDMT1 or TRIM28 foci from three independent experiments (*n* = 3, mean ± SD). (**G**) WB of TRDMT1 and TRIM28 and the survival rate of WT cells and TRDMT1 KO U2OS cells with or without siTRIM28 after 2 Gy IR treatment.

To understand how TRDMT1 is regulated via ubiquitination, we tried to identify the E3 ligases. We pulled down TRDMT1 and performed the mass spectrometry analysis to determine its interacting proteins. Among them, we listed all E3 ligases pulled down by TRDMT1 after washing with sodium chloride at either 150 or 300 mM concentration (Figure 2A). In the E3 ligases pool pulled down by TRDMT1, TRIM28 was the only E3 ligase detected under both washing conditions, and the number of peptides was significantly increased compared to the control group (Figure 2A). TRIM28, also named KAP-1, is known to be involved in the upstream of recruitment of repair proteins of DSBs via modulating chromatin relaxation (32). We knocked down TRIM28 and found the ubiquitination level of TRDMT1 was indeed significantly decreased in TRDMT1^WT^ and further decreased in TRDMT1^G155V^ stably expressed TRDMT1 KO 293 cells (Figure 2B), suggesting that TRIM28 is the E3 ligase for TRDMT1. Moreover, the interaction between TRIM28 and TRDMT1 was confirmed using TRIM28 in IP (Figure 2C). GFP did not pull down any TRIM28, confirming that TRIM28 interacts with TRDMT1 specifically. TRIM28 coprecipitated with TRDMT1 both before and after IR, suggesting that the TRIM28–TRDMT1 interaction is not strictly DNA damage dependent. To pursue the putative link between mRNA m^5^C modification and DNA damage response regulated by TRDMT1, we used KillerRed (KR), a light-excitable and superoxide-releasing chromophore, to conditionally generate local DSBs at a genomic locus in U2OS Tet Response Element (TRE) cells (Figure 2D). When a fusion protein of KR and the transcription activator VP16 (TA-KR) is expressed in these cells, it binds to the array and activates transcription locally, upon light activation, TA-KR releases free radicals intensively at the specific locus of the genome and induces DSBs at the locus (15). In this system, TA-KR represents the sites DSBs in the transcriptionally active regions of the genome; tetR-KR represents sites of damage without transcription; TA-cherry represents actively transcribed sites without damage, and tetR-cherry is the maker of TRE integration without damage and active transcription. We found that both TRDMT1 and TRIM28 were preferentially recruited at sites of TA-KR but not at sites of tetR-KR, TA-cherry and tetR-cherry (Figure 2E and F), indicating that TRDMT1 and TRIM28 are enriched at transcribed damage sites. Knocking down TRIM28 in TRDMT1 KO cells did not further sensitize cells to cisplatin and IR compared to single KD or KO of a single gene, indicating that TRDMT1 and TRIM28 are epistatic in the repair of DSBs (Figure 2G).

**Figure 3.**
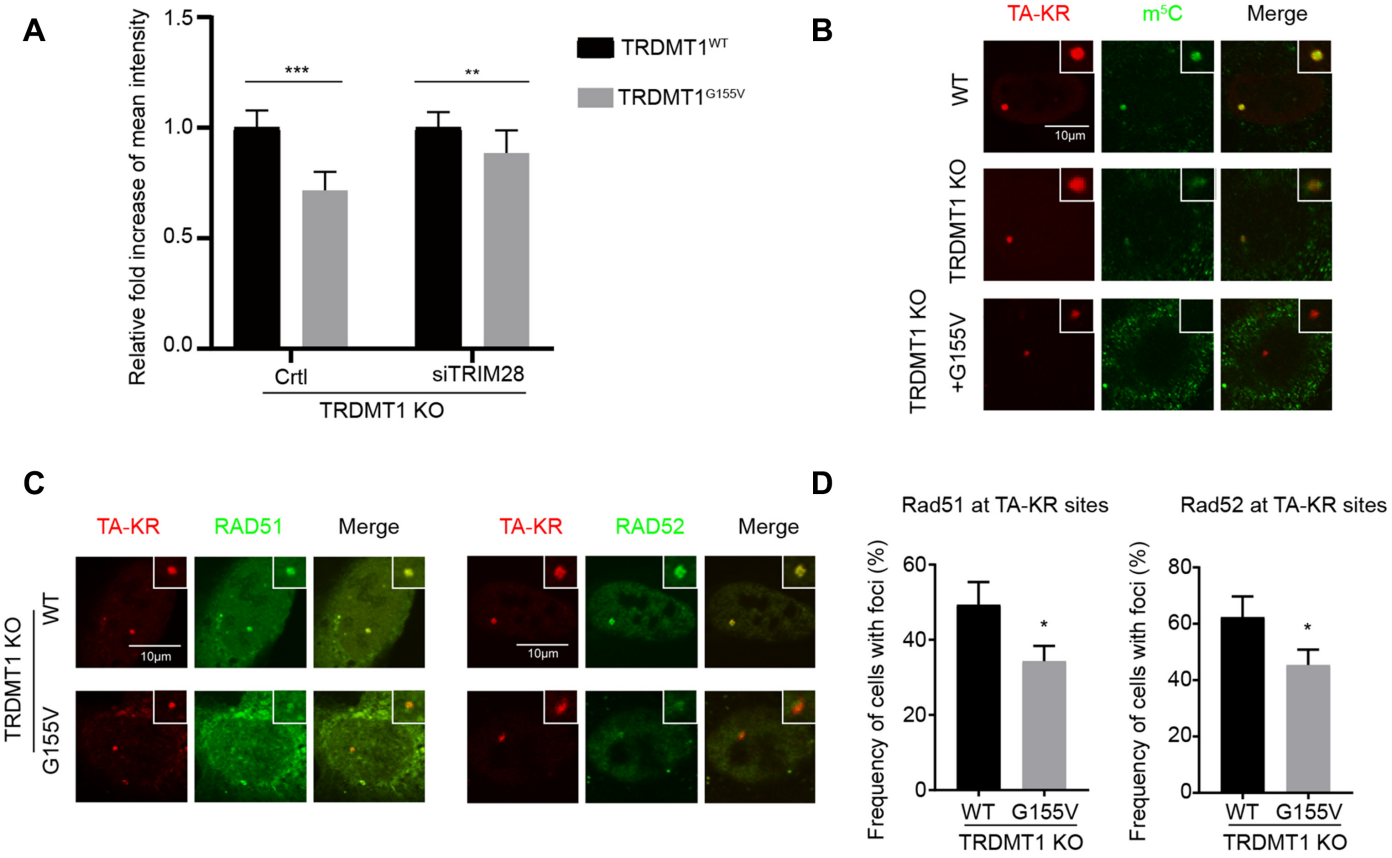
TRDMT1^G155V^ expression diminishes m^5^C and the response of repair factors at damage sites. (**A**) TRDMT1 KO U2OS-TRE cells transfected with TA-KR and TRDMT1^WT^or GFP-TRDMT1^G155V^after pre-treated cells with or without siTRIM28, following with treatment described in Figure 2D. The fold increase of intensity of GFP-TRDMT1 ^WT^ or GFP-TRDMT1^G155V^ at sites of TA-KR was shown (*n* = 3, 10 cells per replicate, mean ± SD). (**B**and**C**) TRDMT1 KO U2OS cells were transfected with TA-KR and Myc-TRDMT1^WT^/TRDMT1^G155V^ following with treatment with 2D and stained for m^5^C (B) and RAD51 or RAD52 (C). (**D**) Quantification of recruitment of RAD51 and RAD52 at TA-KR sites (*n* = 3, 50 cells per replicate, mean ± SD).

Having shown that both TRDMT1 and TRIM28 are recruited to damage sites of the transcriptionally active regions of the genome, we examined the damage response of the TRDMT1^G155V^ mutant. The TRDMT1^G155V^ foci intensity was significantly decreased at the TA-KR site in TRDMT1 U2OS-TRE cells compared to TRDMT1^WT^(Figure 3A, left panel). This is reasonable since the TRDMT1^G155V^ mutant degraded much faster in cells (Figure 1D and E). To understand if depletion of TRIM28-mediated poly ubiquitination contributes to could restore TRDMT1 G155V intensity, we examined the damage response of TRDMT1 G155V after siTRIM28 transfection (Figure 3A, right). In siTRIM28 transfected cells, the intensity of G155V is about 80–90% of WT TRDMT1 at sites of damage, whereas in WT cells the intensity of G155V is only 60% of WT TRDMT1. Thus, the accumulation of G155V at DNA damage sites is partially restored in siTRIM28-treated cells. The slight reduction of TRDMT1 G155V intensity in siTRIM28 cells (around 10–20% of WT) might be dependent on incomplete TRIM28 knockdown or an alternative but inefficient ubiquitin ligase for TRDMT1. Since TRDMT1 is a ‘writer’ of m^5^C in mRNA during the DNA damage response, not surprisingly, TRDMT1^G155V^ failed to restore the local m^5^C level in U2OS-TRE (Figure 3B). Notably, in contrast to TRDMT1^WT^, TRDMT1^G155V^ failed to restore the recruitment of RAD51 and RAD52 at damage sites, which are critical factors of TC-HR, at TA-KR sites in U2OS-TRE TRDMT1 KO cells (Figure 3C and D). These results indicate that the complete response to Cisplatin treatment of TRDMT1^G155V^ bearing patient might due to defective DNA repair for DSBs.

### Ubiquitination of TRDMT1 at K251 is required for cell survival after DNA damage

**Figure 4.**
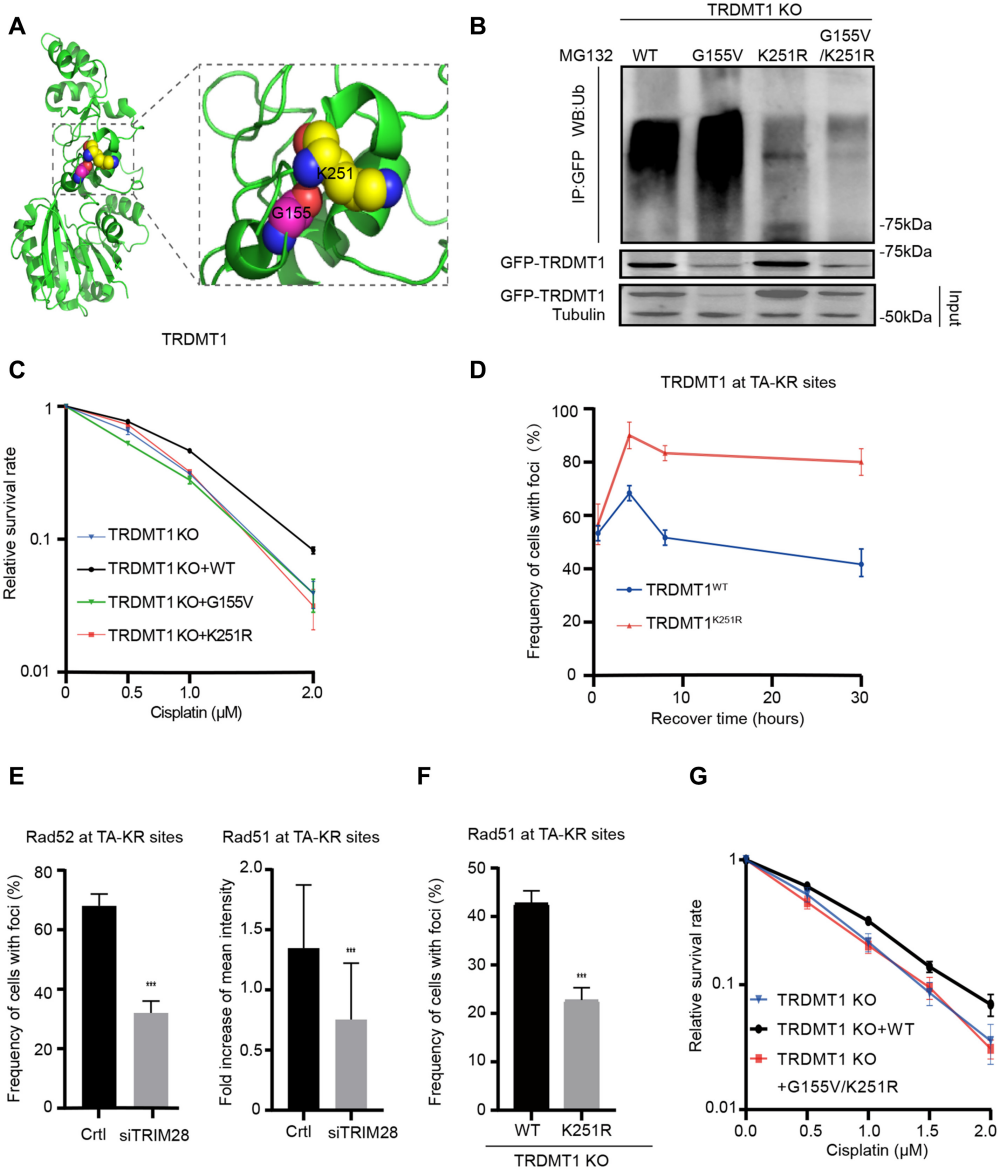
Ubiquitination of TRDMT1 at its K251 is required for cell survival. (**A**) 3D structure of TRDMT1 using PyMOL marked with glycine 155 (G155) and its adjacent lysine 251 (K251). (**B**) 293 TRDMT1 KO cells were transfected with GFP-tagged TRDMT1^WT^, TRDMT1^G155V^, TRDMT1^K251R^ and TRDMT1^G155V+K251R^ mutants, respectively. Cells were pulled with anti-GFP and immunoblotted with anti-Ubi. (**C**) The survival rate of U2OS-TRE TRDMT1 KO cells and U2OS-TRE TRDMT1 KO cells transfected with TRDMT1^WT^, TRDMT1^G155V^, TRDMT1^K251R^ after cisplatin treatment at the indicated dose. (**D**) TRDMT1 KO U2OS cells were transfected with TA-KR and GFP-TRDMT1^WT^/GFP-TRDMT1^K251R^ were exposed to light for 30 min for KR activation and allowed to recover for indicated times (30 min, 4 h, 8 h, 30 h) before fixation. The frequency of cells with GFP-TRDMT1 at TA-KRsites was quantified. (**E**) Quantification of RAD52 (*n* = 3, 50 cells per replicate, mean ± SD) and RAD51 at TA-KR sites (*n* = 20, mean ± SD) after pretreated cells with siTRIM28 or siCtrl. (**F**) TRDMT1 KO U2OS cells were transfected with TA-KR and FLAG-HA-TRDMT1^WT^/ FLAG-HA-TRDMT1^K251R^ following with treatment with 2D and stained for RAD51. The frequency of RAD51 at TA-KR sites was quantified (*n* = 3, 50 cells per replicate, mean ± SD). (**G**) The survival rate of U2OS-TRE TRDMT1 KO cells and U2OS-TRE TRDMT1 KO cells transfected with TRDMT1^WT^, TRDMT1^G155V^/^K251R^ after cisplatin treatment at the indicated dose.

The ubiquitin must be attached to a lysine site of a substrate. Given that TRIM28 co-localizes and is required poly-ubiquitination TRDMT1, we tried to determine the ubiquitination site of TRDMT1. There are 25 lysine residues in TRDMT1. The fact that TRDMT1^G155V^ is highly ubiquitinated prompts us to search any lysine residues near the glycine 155. By analyzing the crystal structure of TRDMT1 from the database (uniprot.org), we found that lysine 251 is the nearest lysine site to glycine 155 (Figure 4A). We constructed GFP-tagged TRDMT1^WT^, TRDMT1^G155V^, TRDMT1^K251R^ and TRDMT1^G155V/K251R^ mutants and transfected them into TRDMT1 KO U2OS-TRE cells. We found that both TRDMT1^K251R^ and TRDMT1^G155V/K251R^ exhibited decreased ubiquitination of TRDMT1 (Figure 4B). In contrast, mutation of another lysine 122, K122R, which is located far away from G155, did not alter the level of ubiquitination of TRDMT1 (Supplementary Figure S1A).

To confirm the role of K251 mediated polyubiquitination in DNA repair, we measured the cell survival after damage. TRDMT1^K251R^ expressed cells were as sensitive as TRDMT1 KO cells to cisplatin compared to TRDMT1^wt^ expressed TRDMT1 KO cells (Figure 4C and Supplementary Figure S1B). Thus, either hyper-ubiquitination or loss of ubiquitination of TRDMT1 could sensitize the cells to DNA damage. We also found that TRDMT1^K251R^ was recruited at sites of TA-KR as well as TRDMT1^WT^; however, TRDMT1^K251R^ was recruited to sites of TA-KR more efficiently and remained for a long time (Figure 4D), indicating that the remaining of TRDMT1^K251R^ might affect repair progression. Interestingly, siTRIM28 also decreased the recruitment of Rad52 and Rad51 at TA-KR sites (Figure 4E), indicating that level of TRDMT1 at sites of DNA damage is tightly regulated. Given RAD51 foci is the critical step for repair progression, we measured the effect of K251R mutation on Rad51 foci. K251R mutation compromised the frequency of Rad51 foci (Figure 4F). To understand the effects of the G155V/K251R double mutant on DNA repair, we measured cell survival after cisplatin treatment. If the G155V/K251R double mutant is defective for repair, the TRDMT1 KO cells expressing this mutant should remain sensitive to DNA damage. Indeed, expression of the G155V/K251R double mutant in TRDMT1 KO cells did not rescue cell survival in cisplatin (Figure 4G). Together, poly-ubiquitination mediated TRDMT1 degradation is required for repair progression and cell survival.

### Reduced TRDMT1 expression increases cisplatin sensitivity in tumors

**Figure 5.**
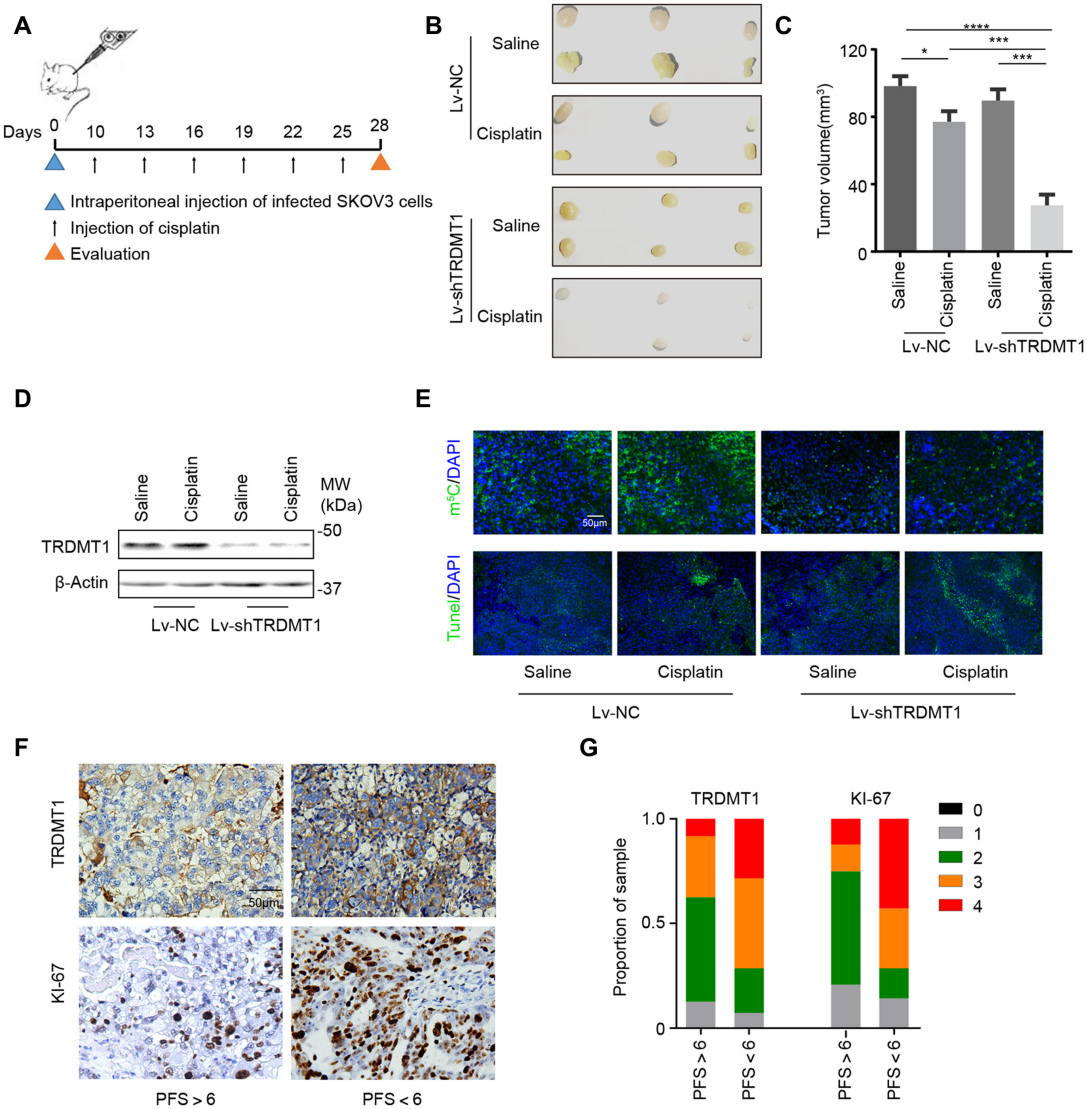
Reduced TRDMT1 expression renders cisplatin sensitization *in vivo*. (**A**) Lv-shTRDMT1 or Lv-NC transfected SKOV3 cells were subcutaneously injected into BALB/c nude mice, a week after injection, cisplatin (5 mg/kg) or saline was injected into the center of the xenograft tumors twice per week for three consecutive weeks. (**B**) Representative images of tumors in xenografts transfected with LV-shTRDMT1/LV-NC with saline or cisplatin treatment. (**C**) Tumor size of SKOV3 subcutaneous xenograft tumors (*n* = 6, mean ± SD). (**D**) WB for TRDMT1 in tumor tissues collected from mice in each group (tissues from six tumors in the same group were mixed into one sample). (**E**) Representative photographs of m5C staining and TUNEL analysis in tumors treated as indicated above. (**F**and**G**) IHC of TRDMT1 and KI-67 expression in ovarian cancer tissues of patients who were platinum-sensitive (PFS > 6) and -resistant (PFS < 6) (F). Staining was assessed and scored on a scale of 0 (<5% staining) to 4 (>75% staining) in (G). Quantification of IHC staining (*n* = 38, PFS > 6, *n* = 24; PFS < 6, *n* = 14) was shown.

Given that TRDMT1 suppression leads to increased cisplatin and IR sensitivity in cells, we validated the role of TRDMT1 in drug resistance *in vivo*, using a model of epithelial ovarian cancer (EOC) by subcutaneous injection of SKOV3 cells that were infected with lentiviruses (LV) carrying TRDMT1-RNAi (Lv-shTRDMT1) or NC-RNAi (Lv-NC) was established (Figure 5A). There were no significant differences in tumor size between TRDMT1-depleted tumors and the control tumors. However, with cisplatin treatment, xenografts developed from Lv-shTRDMT1 infected SKOV3 cells grew statistically smaller (Figure 5B–D). In Lv-shTRDMT1 infected tumor tissue, we observed an overall decrease of m^5^C (Figure 5E). These results suggesting TRDMT1 depletion sensitizes EOC cells to cisplatin, which correlates to the level of m^5^C. To further explore the correlation between TRDMT1 and drug resistance, we quantified TRDMT1 and a marker of cell proliferation (Ki67) expression in primary EOC specimens derived from 38 EOC patients (Supplementary Table S1). As expected, TRDMT1 protein level and the number of Ki67-positive cells elevated in tumors from patients with progression-free survival (PFS) <6 months (clinically described as platinum-resistant), while their expression was decreased in tumors from patients with PFS >6 months (platinum-sensitive) (Figure 5F and G), supporting that high TRDMT1 expression is correlated to enhanced drug resistance.

### The TRDMT1 Inhibitor YW-1842 reduces m^5^C induction and HR

**Figure 6.**
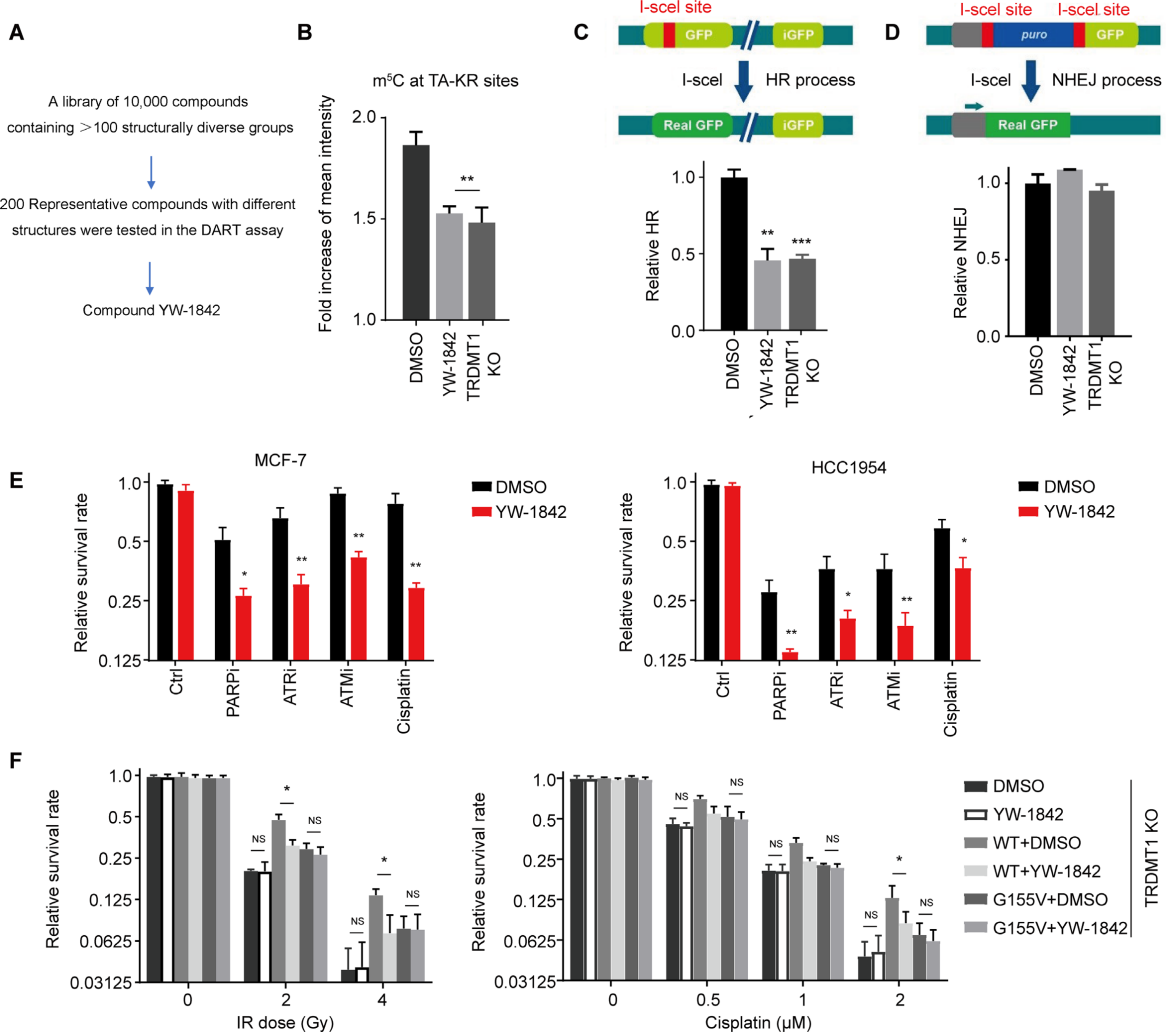
The TRDMT1 Inhibitor YW-1842 specifically sensitizes cells to DNA damage agents. (**A**) A screening scheme of YW-1842. (**B**) SCE WT cells were transfected with TA-KR and were cultured with 2.5 μM/l compound YW-1842 or not for 6 h, m^5^C (*n* = 3, 50 cells per replicated, mean ± SD) was stained and quantified. (**C**and**D**) DR-GFP (C) or EJ5-GFP (D) cells were pre-treated with 2.5 μM/l compound YW-1842 for 6 h or TRDMT1 siRNA for 48 h, or DMSO. Then, the cells were transfected with NLS-I-SceI plasmid to induce DSB. The efficiency of HR (C) or NHEJ (D) was analyzed by flow-cytometry (*n* = 3, mean ± SD). (**E**) After seeding, MCF-7, HCC1954 and HCC1937 cells were pre-treated with or without 2.5 μM/l compound YW-1842 for 6 h, then cultured with chemotherapy drugs (1 μM/l cisplatin; 1 μM/l ATRi AZ20; 10 μM/l ATMi KU55933; 1 μM/l PARPi Olaparib) for 10 days. The survival rate of these cells was analyzed (*n* = 3, mean ± SD). (**F**) TRDMT1 KO U2OS cells were transfected with GFP-TRDMT1^WT^ or GFP-TRDMT1^G155V^ with or without 2.5 μM/l compound YW-1842, the survival rate of these cells after IR or cisplatin was shown (n = 3, mean ± SD).

Under normal conditions, TRDMT1 transfers the methyl group from SAM to the fifth carbon atom of cytosine on RNA to produce m^5^C and SAM was converted to SAH (4). Previous studies and our current findings (Figures 1 and 5) as described above suggested TRDMT1 is a promising target for cancer therapy based on its role in DNA repair (3). To identify a small molecule TRDMT1 inhibitor, an in-house compound library was screened, and a promising hit was discovered. Subsequent SAR optimization led to the identification of compound YW-1842 (detailed medicinal chemistry works will be published in due course), which exhibited -m^5^C inhibitory activity almost equivalent to TRDMT1 depletion at the concentration of 2.5 μM/L (Figure 6A and B). To rule out the possibility that YW-1842 is a cytotoxic drug, the antiproliferative effect of YW-1842 was measured and the compound did not prohibit cell proliferation at the concentration below 5 μM/l (Supplementary Figure S2A). DNA repair efficiency was also decreased with the compound as evidenced by a delayed clearance of γ-H2AX foci in cells after IR (Supplementary Figure S2B) with 2.5 μM/l YW-1842. Next, we used reporter assays to confirm the effects of YW-1842 on the HR-mediated repair of nuclease-generated DSBs at transcribed loci. Using the DR-GFP assay, we confirmed that YW-1842 diminished the repair of I-SceI-generated DSBs in comparison with the DMSO treated group as well as siTRDMT1 (Figure 6C). However, nonhomologous end joining (NHEJ) showed no obvious changes in either TRDMT1 KD cells or the TRDMT1 wild-type cells treated by compound YW-1842 (Figure 6D). This observation is in line with our previous findings that TRDMT1 loss would not affect the efficiency of NHEJ (16). These data implicates YW-1842 is a potent inhibitor for TRDMT1.

### YW-1842 sensitizes tumor cells to DNA damage in a TRDMT1-dependent manner

We have demonstrated that TRDMT1 plays a pivotal role in DNA repair by the HR pathway (16). Therefore, the inhibition of TRDMT1 activity may augment the cytotoxic effects of radiation and the DNA damaging agents, e.g. H_2_O_2_; cisplatin; ATR inhibitor (ATRi) AZ20; ATMi KU55933; PARPi Olaparib. These DNA damaging agents cause comprehensive lesions, while DSBs accumulated by these agents are lethal to cells and could be repaired by the HR pathway (29,30,33,34). Importantly, compound YW-1842 at 2.5 μM/l, which does not kill cells alone (Supplementary Figure S2A), sensitizes the cells to above DNA damaging agents compared to single treatment (Figure 6E). Especially, YW-1842 at 2.5 μM demonstrated a strong synergy with a marketed drug PARPi Olaparib at 1 μM in a triple-negative breast cancer HCC 1954 cell lines (Figure 6E). Moreover, when YW-1842 was combined with IR, a clear sensitivity enhancement for IR was exhibited (Supplementary Figure S3). The combination of YW-1842 with H_2_O_2_ also decreased the survival rate of all three cell lines compared to DMSO (Supplementary Figure S3). Finally, we tested the effect of YW-1842 after damage in TRDMT1 KO cells expressing TRDMT1^WT^ or TRDMT1^G155V^. Compound YW-1842 does not sensitize TRDMT1 KO, while TRDMT1^WT^ expression rescued the sensitivity of TRDMT1 KO cells to either IR or cisplatin, indicating that the effect of YW-1842 is dependent on TRDMT1 expression (Figure 6F). Consistently, TRDMT1^G155V^ expression did not rescue the sensitivity of TRDMT1 KO cells to either IR or cisplatin, supporting our findings that the depletion of TRDMT1 due to TRDMT1^G155V^-induced hyper ubiquitination renders the drug sensitivity in the patient (Figure 6F). These findings strengthen the notion that concurrent modulation of TRDMT1 may be a therapeutically effective adjunctive treatment with chemotherapy or radiotherapy for patients who harbor TRDMT1-upregulated tumors.

## DISCUSSION

Our data demonstrate that the loss of TRDMT1 expression in cancer could lead to increased sensitivity of cancer cells to chemotherapy and/or radiotherapy due to inefficient DNA repair. The markedly reduced overall m^5^C methylation in RNA and changes of the DNA damage response in TRDMT1 KO cells could be rescued by stable expression of TRDMT1^WT^ but not the patient-derived mutant TRDMT1^G155V^. This phenomenon was likely due to the loss of TRDMT1 via hyper-ubiquitination. When DNA damage is induced in the transcribed regions in the genome, R loops are efficiently induced (16). Our previous study shows that the DNA: RNA hybrids around SSBs and DSBs may recruit TRDMT1 and serve as its substrate. Once modified with m^5^C, the DNA: RNA hybrids recruit RAD52 and RAD51 more efficiently to promote HR and protect cells from DNA damage (16). In this study, we identified that the E3 ligase TRIM28 is recruited by TRDMT1 and targets its lysine K251 for poly-ubiquitination, which is likely required for removal of TRDMT1 from DNA damage site and completion of repair. Although K251 is likely the primary site of ubiquitination, it may not be the only site. Indeed, we observed low levels of ubiquitination of the K251R mutant. Both K251R and G155V/K251R mutants are ubiquitinated at low levels (Figure 4B). This is very different from the G155V mutant, which is increasingly ubiquitinated compared to WT (Figure 4B). These results argue that the effects of G155V on ubiquitination are largely, if not completely, dependent on K251, supporting the notion that G155V affects K251 ubiquitination. However, we agree with the reviewer that the effect of G155V may not exclusively be attributed to the change of local folding near K251. We cannot rule out the possibility that G155V affects the folding of TRDMT1 extensively and makes it a better substrate of ubiquitination. Interestingly, both loss of TRDMT1 ubiquitination (e.g. TRIM28 loss) and TRDMT1 hyper ubiquitination (e.g. G155V) impair DNA repair and increase cellular sensitivity to DNA damage. It is worth pointing out that the expression of the G155V/K251R double mutant in TRDMT1 KO cells did not rescue cell survival in cisplatin (Figure 4G). This result suggests that the loss of K251 ubiquitination impairs repair, and this effect is dominant over that of G155V. It is likely that the TRDMT1 G155V/K251R mutant cannot be removed from DNA damage sites properly, which blocks the completion of repair. Together, our data suggest that the amount of TRDMT1 at sites of DNA damage has to be tightly regulated in cells to ensure efficient repair and cell survival. Both the lack of TRDMT1 and the failure in TRDMT1 removal are detrimental to DNA repair.

TRDMT1 was known to modify tRNA in the cytoplasm (8,35); TRDMT1-dependent m^5^C sites has also been detected in mRNAs (9–11); biochemical fractionation experiments in Drosophila showed that TRDMT1 was mostly a cytoplasmic protein with a minor nuclear fraction tightly attached to the nuclear matrix (36); TRDMT1 was shown to exhibit nucleo-cytoplasmic shuttling in response to cellular stress (37). Previously, we showed TRDMT1 carrying nuclear localization signal (NLS) localized to damage sites in the nucleus and restored m^5^C formation and γ-H2AX clearance at the locus marked by TA-KR, supporting that the nuclear localization of TRDMT1 is important for DNA repair (14,22). In addition to RAD51, the repair of TA-KR-induced DSBs requires RAD52 (14). Here, we found TRDMT1^G155V^ expression led to reduced mRNA m^5^C methylation and inefficient HR as evidenced by decreased recruitment of RAD51 and RAD52, and delayed clearance of γ-H2AX foci at sites of DNA damage induced by TA-KR or IR compared to TRDMT1^WT^ transfection. Thus, the G155V mutation significantly reduces the function of TRDMT1 in the face of DNA damage, which may weaken the function of TDRMT1 in DNA repair, increasing the effects of chemotherapeutic drugs or radiation. It is reasonable to speculate that additional cancer mutations that stabilize TRDMT1 may also enhance the therapeutic response in cancers.

E3s control both the efficiency and substrate specificity of the ubiquitination reaction (38). Here, we found that TRIM28 is the E3 ligase of TRDMT1. The TRIM28 not only mediates the ubiquitination of a particular protein, but also mediates other biological processes, such as DNA damage repair, repression of transcriptional elongation, and cancer development (39–41). In ovary cancer, TRIM28 high expression was an independent predictor for patients with ovarian cancer (42). It is also shown that TRIM28 plays a unique and essential role in transcriptional elongation (43). We show that TRIM28 is specifically recruited to damage sites where TRDMT1 is enriched, indicating the unique role of TRIM28 in protecting the transcriptionally active regions of the genome from damage. It would be interesting to investigate whether TRIM28 ubiquitinates and removes other repair proteins to complete repair and whether the expression of TRIM28 affects the therapeutic response of cancers.

This intriguing feature of TRDMT1 in DNA damage regulation pushed us to further explore the possibility of discovering TRDMT1 small molecule inhibitors. From an extensive drug discovery campaign, we identified a highly potent inhibitor YW-1842. We showed that YW-1842 efficiently inhibited TRDMT1 recruitment, m^5^C formation and HR efficiency at DNA damage sites, and enhanced the cellular sensitivity to multiple DNA-damaging agents. These findings further supported the notion that TRDMT1 is an attractive target to sensitize cancer cells to chemotherapy.

Importantly, TRDMT1 is upregulated in a subset of tumors and correlated with the poor prognosis of patients. Some selective inhibitors of specific protein methyltransferases, including DOT1L, EZH2 and PRMT5, are currently under clinical investigation as cancer therapeutics (20,21,44). However, the structural details of the various RNMTs are unique and distinguished from those of the protein methyltransferases that have been proved quite amenable to modulation by small-molecule inhibitors with good pharmacological properties (45). YW-1842 demonstrated high inhibitory activity, providing a starting point for further optimization for therapeutic purposes. Taken together, our study demonstrated that the expression, degradation, and activity of TRDMT1 are highly relevant to the response of cancer cells to chemotherapy, and made an important step toward the synthesis of potent, specific inhibitors for TRDMT1, providing a basis for improving chemotherapy in future studies.

## DATA AVAILABILITY

The datasets generated and/or analyzed during the current study are available from the corresponding author upon request. The LC/MS raw files and search results were deposited into MassIVE data repository. https://secure-web.cisco.com/121wrFVgr3JpWPCftV2ydTxoo0OZPkb5S3YEfEQ-brgqsZa0ZDX41MctPmYVOWjwTlhps_Qf8T5T0qwOC WdZx9BsU11aWsS_jvrnvF21ph1w9SSlBq_vTaDYbyp-S46DQru_4TPIV-vDrPafflUtLk5DKVT64fS3-Vq7llQgt_sP7XQGGrIgghPI68i04Ofx9m2Yd8jfUaOeWX Xod8CIs5bv9crWyuLoRdvjKV_0SWjrmDcjy-37FcQOF8IBaQKKL/https%3A%2F%2Fmassive.ucsd.edu%2FProteoSAFe%2Fstatic%2Fmassive.jsp Accession number: MSV000085313

## Supplementary Material

zcab010_Supplemental_File
