# Supplementary material for: Ubiquitination-mediated degradation of TRDMT1 regulates homologous recombination and therapeutic response
Source: NAR Cancer. 2021 Mar 22;3(1):zcab010. doi: 10.1093/narcan/zcab010 (PMC7984809; doi:10.1093/narcan/zcab010)
Supplement: zcab010_Supplemental_File [file zcab010_supplemental_file.pdf]

Supplementary Figures and legends

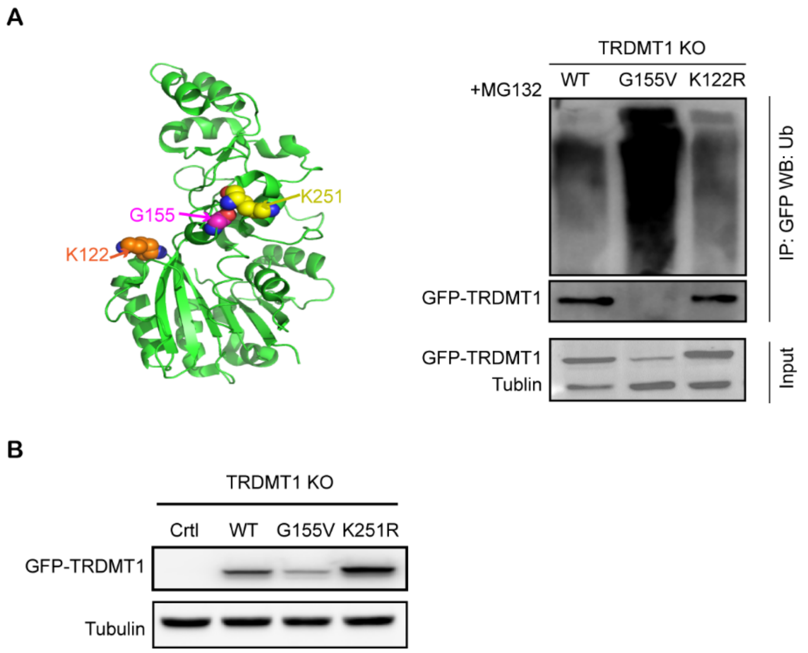

Figure S1 |Lysine K122R mutant did not alter the level of ubiquitination of TRDMT1 (A)3D structure of TRDMT1(PyMOL) marked with glycine 155(G155) and its adjacent lysine 251 (K251) and other lysine 122 (K122). 293 TRDMT1 KO cells were transfected with GFP-tagged TRDMT1^{WT}, TRDMT1^{G155V}, or TRDMT1^{K122R}. Cells were pulled with anti-GFP and immunoblot with anti-Ub. (**B**) WB of the U2OS cells transfected with TRDMT1 mutants in Fig. 4C.

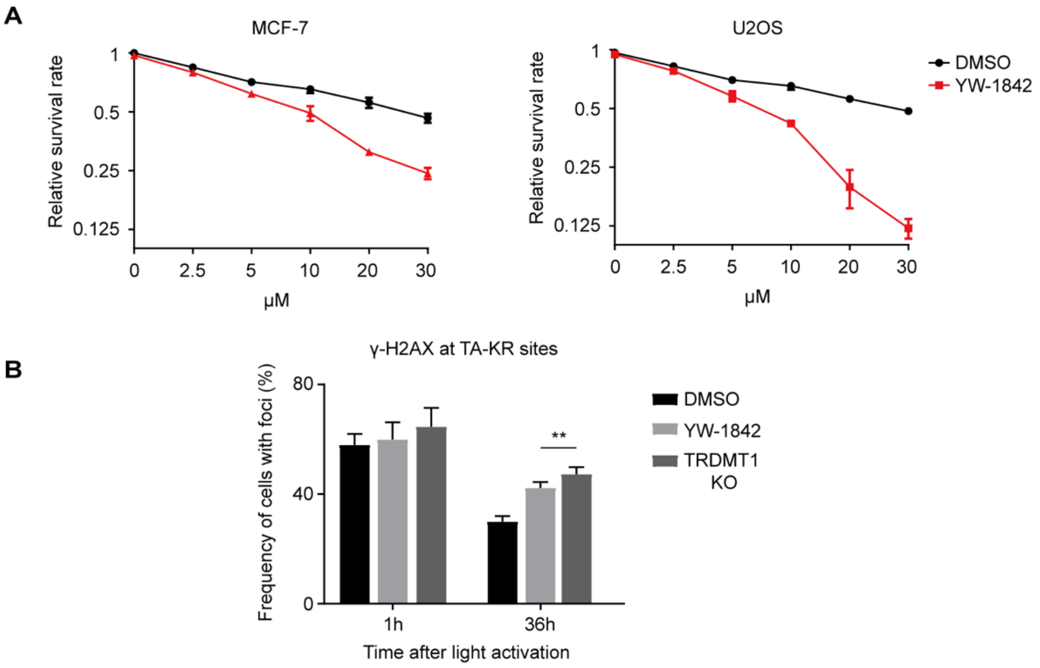

Figure S2 | TRDMT1 inhibitor YW-1842 delays the repair of DSBs at the transcribed genome.

(**A**) U2OS or MCF7 cells were treated with or without compound YW-1842 at the indicated concerntration. The survival rate of these cells was shown (n = 3, Mean ± SD). (**B**) SCE WT cells were transfected with TA-KR and were cultured with 2.5μM YW-1842 or not for 6 hours, γ-H2AX (n=3, 50 cells per replicated，Mean ± SD) were stained and quantified.

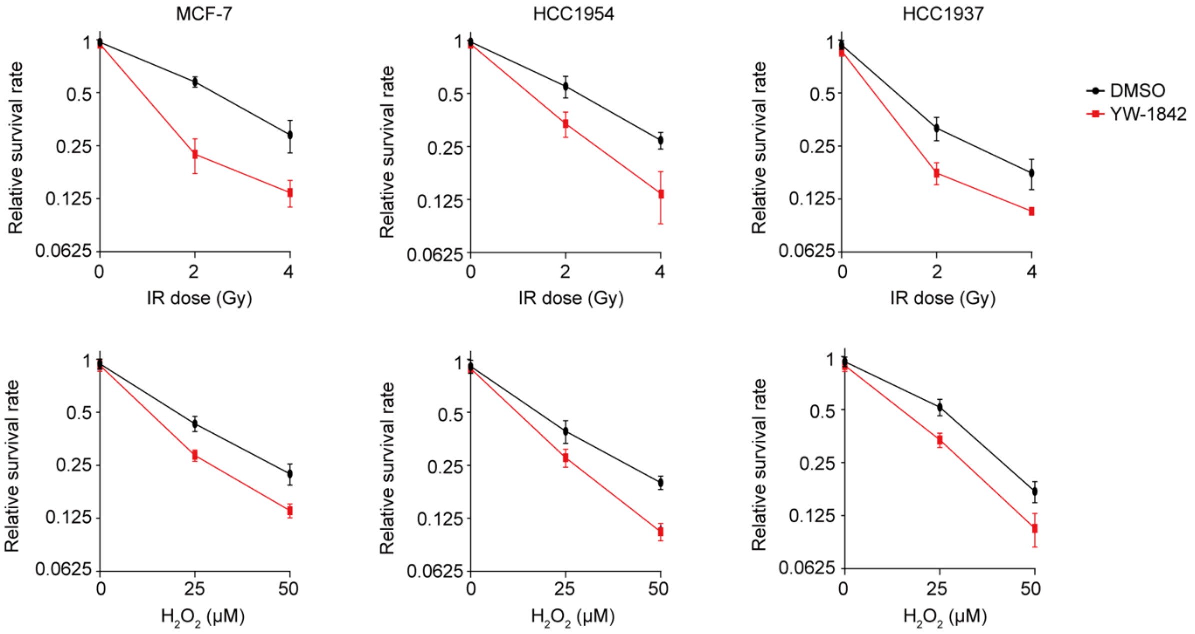

Figure S3 | TRDMT1 inhibitor YW-1842 sensitizes tumor cells to IR and H_2O_2.

MCF-7, HCC1954, and HCC1937 cells were pre-treated with or without TRDMT1 inhibitor YW-1842 (2.5μM) for 6hrs, then the cells were exposed to IR or H2O2, and cultured for 7-14 days. The survival rate of these cells was analyzed (n = 3, Mean ± SD)

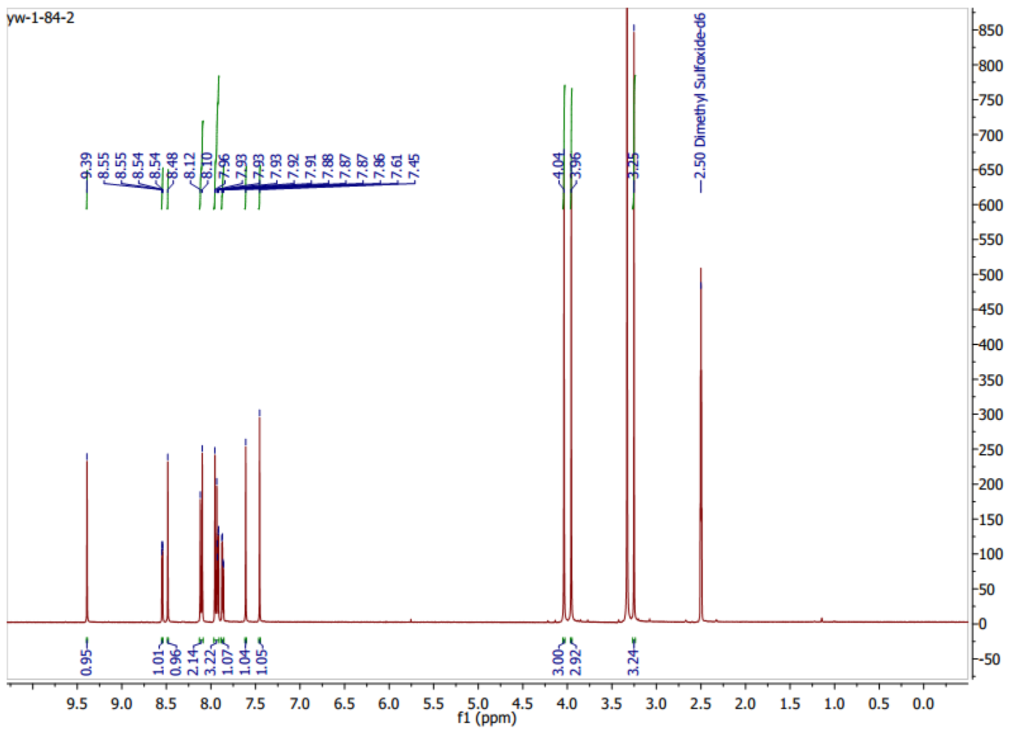

Figure S4| ^{1}H NMR spectra of YW-1842.

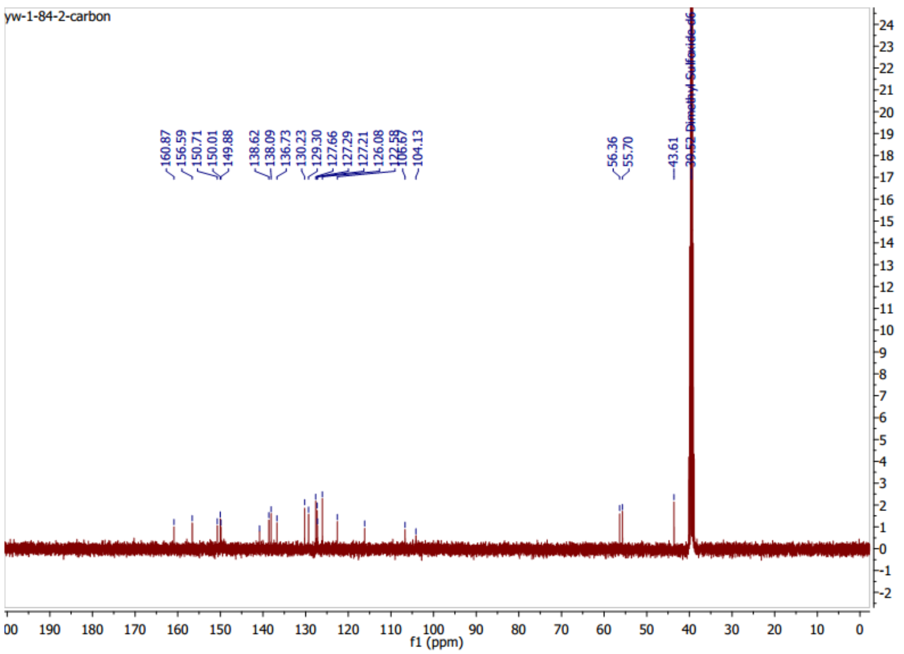

Figure S5 | ^{13}C NMR spectra of YW-1842.

Supplementary Table S1. Clinicopathological characteristics of EOC patients (n= 38)

		PFS＞6 (n=24)	PFS＜6 (n=14)
Age(yrs)		57.13±8.65	56.86±6.85
Stage	IIIC	19	12
	Ⅳ	5	2
Grade	High	2	1
	Moderate	14	9
	Low	8	4

Supplementary Table S2. Sequences of PCR Primers

Primer	Sequence
TRDMT1-F-EcoRI	CCGGAATTCTGATGGAGCCCCTGCGGGTG
TRDMT1-R-BamH1	CGCGGATCCTTATTCATATAAGATTTTGATTAG
TRDMT1 K122R-F	CTTTTGGAAAATGTTAGAGGTTTTGAAGTATCT
TRDMT1 K122R-R	AGATACTTCAAAACCTCTAACATTTTCCAAAAG
TRDMT1 K251R-F	AGTGATCTCTCTGTGAGAATGCTAAAAGATTTT
TRDMT1 K251R-R	AAAATCTTTTAGCATTCTCACAGAGAGATCACT
TRDMT1 G155V-F	TTATCTCCAACCTCTCTTGTCATTCCAAATTCAAGGCTAC
TRDMT1 G155V-R	GTAGCCTTGAATTTGGAATGACAAGAGAGGTTGGAGATAA

Supplementary methods

Synthesis and characterization of YW-1842

All starting materials and reagents were either obtained from commercial suppliers or prepared according to literature reported procedures. All purchased chemicals and solvents were used without further purification unless otherwise noted. Flash chromatography was performed using silica gel (300−400 mesh) on Teledyne ISCO CombiFlash Rf. All reactions were monitored by thin-layer chromatography (TLC) or liquid chromatography–mass spectrometry (LC-MS) (Thermo Finnigan LCQ Deca with Thermo Surveyor LCMS System) running a gradient of increasing Methanol (30% to 100%) in water both containing 0.1% formic acid at 0.5 mL/min on a short path C-18 reverse phase column. ^{1}H NMR spectral data were recorded on Varian 400 NMR spectrometer, and ^{13}C NMR was recorded on Varian Mercury 101 NMR spectrometer at ambient temperature. Chemicals shifts (δ) were reported in parts per million, coupling constants (*J*) values were in hertz, and the splitting patterns were abbreviated as follows: s for singlet; d for doublet; t for triplet; q for quartet; and m for multiplet. Microwave reactions were carried out using a Biotage Initiator EXP microwave synthesizer. Reaction temperature was monitored by the internal probe.

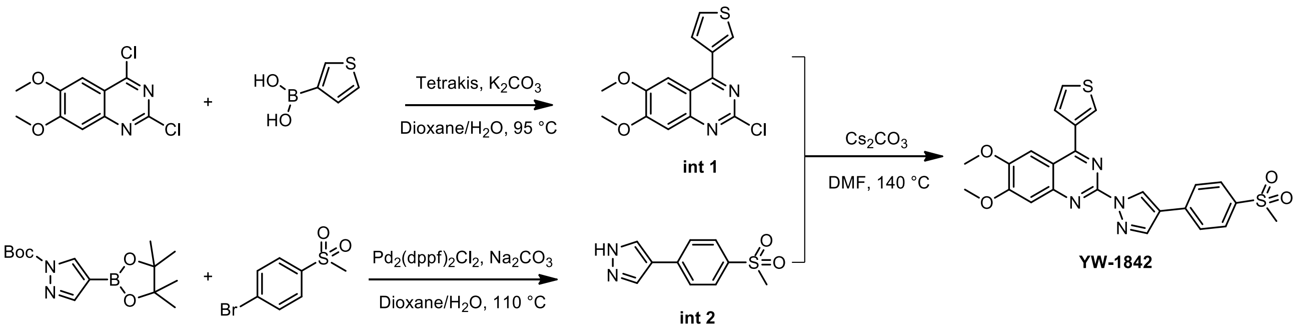

A mixture of 2,4-dichloro-6,7-dimethoxyquinazoline (1.0 mmol), thiophen-3-ylboronic acid (1.0 mmol), and K_2CO_3 (2.0 mmol) in dioxane (4.0 mL) and water (1.0 mL) were degassed by N_2 for 10 min. $Pd(PPh_3)_4$ (0.03 mmol) was added in one portion and the mixture was degassed for another 5 min. The resulted suspension was stirred at 95 °C under N_2 atmosphere for 16h. The reaction mixture was allowed to cool to room temperature and separated between ethyl acetate and water. The organic layer was washed with water and brine, dried over sodium sulfate and concentrated to run column (Hexane/ethyl acetate=4/1) to get **int 1** as a white

solid (yield 63%). ^{1}H NMR (400 MHz, $CDCl_3$) δ 7.96 (d, J = 3.0 Hz, 1H), 7.63 (d, J = 5.0 Hz, 1H), 7.54 (dd, J = 5.0, 3.0 Hz, 1H), 7.49 (s, 1H), 7.31 (s, 1H), 4.06 (s, 3H), 3.98 (s, 3H). LC-MS: $[M+H]^+$ 307.2.

A mixture of tert-butyl 4-(4,4,5,5-tetramethyl-1,3,2-dioxaborolan-2-yl)-1H-pyrazole-1-carboxylate (3.4 mmol), 1-bromo-4-(methylsulfonyl)benzene (3.3 mmol), and Na_2CO_3 (2.0 mmol) in dioxane (20.0 mL) and water (5.0 mL) were degassed by N_2 for 10 min. Pd(dppf)$_2$Cl$_2$ (0.03 mmol) was added in one portion and the mixture was degassed for another 10 min. The resulted suspension was stirred at 120 °C under N_2 atmosphere for 16h. The reaction mixture was allowed to cool to room temperature and separated between ethyl acetate and water. The organic layer was washed with water and brine, dried over sodium sulfate and concentrated to run column (DCM/MeOH=20/1) to get **int 2** as a beige solid (yield 58%). ^{1}H NMR (400 MHz, DMSO-d_6) δ 13.14 (s, 1H), 8.40 (s, 1H), 8.08 (s, 1H), 7.87 (s, 4H), 3.20 (s, 3H). LC-MS: $[M+H]^+$ 223.1.

A mixture of **int 1** (0.2 mmol), **int 2** (0.2 mmol), and Cs_2CO_3 (0.3 mmol) in DMF (1.0 mL) was heated to 140 °C under microwave irradiation for 1h. The reaction mixture was concentrated to run column (DCM/MeOH=20/1) to get **YW-1842** as a beige solid (yield 33%). ^{1}H NMR (400 MHz, DMSO-d_6) δ 9.39 (s, 1H), 8.55 (dd, J = 2.8, 1.2 Hz, 1H), 8.48 (s, 1H), 8.11 (d, J = 8.5 Hz, 2H), 7.97 – 7.91 (m, 3H), 7.87 (dd, J = 5.0, 2.9 Hz, 1H), 7.61 (s, 1H), 7.45 (s, 1H), 4.04 (s, 3H), 3.96 (s, 3H), 3.25 (s, 3H). ^{13}C NMR (101 MHz, DMSO-d_6) δ 160.87, 156.59, 150.71, 150.01, 149.88, 140.82, 138.62, 138.09, 136.73, 130.23, 129.30, 127.66 (2C), 127.29, 127.21, 126.08 (2C), 122.58, 116.16, 106.67, 104.13, 56.36, 55.70, 43.61. LC-MS: $[M+H]^+$ 493.4 (Fig. S4 and Fig. S5).
